# Preliminary study on the protective effect of remazolam against sepsis-induced acute respiratory distress syndrome (ARDS)

**DOI:** 10.7717/peerj.17205

**Published:** 2024-04-18

**Authors:** Xiaoxin Gao, Rujun Zhang, Zhenzhou Wang, Qingan Chen, Zhenlin Lei, Yanan Yang, Jia Tian

**Affiliations:** 1 Intensive Medical Unit, Hainan General Hospital, Hainan Affiliated Hospital of Hainan Medical University, Haikou, China; 2 Department of Cardiology, Hainan Province Clinical Medical Center, Hainan General Hospital; Hainan Affiliated Hospital of Hainan Medical University, Haikou, China; 3 Department of Emergency Medicine, Hainan Cancer Hospital, Haikou, China

**Keywords:** Sepsis, Inflammatory factors, ARDS, Remazolam, HMGB1, Immune function

## Abstract

**Background:**

Sepsis can disrupt immune regulation and lead to acute respiratory distress syndrome (ARDS) frequently. Remazolam, a fast-acting hypnotic drug with superior qualities compared to other drugs, was investigated for its potential protective effects against sepsis-induced ARDS.

**Methods:**

Forty Sprague-Dawley rats were randomly divided into four groups, including the sepsis + saline group, sham operation + saline group, sham operation + remazolam group and the sepsis + remazolam group. Lung tissues of rats were extracted for HE staining to assess lung damage, and the wet weight to dry weight (W/D) ratio was calculated. The levels of proinflammatory factors, anti-inflammatory factors, CD4+ and CD8+ T cells in peripheral blood, MDA, MPO, and ATP in the lung tissue were measured by using ELISA. Western blotting was performed to determine the protein expression of HMGB1 in lung tissues.

**Results:**

In comparison to the sham operation + saline and sham operation + remazolam groups, the sepsis + saline group exhibited significantly higher values for W/D ratio, lung damage score, IL-1β, IL-6, TNF-α, PCT, CRP, MDP and MPO, while exhibiting lower levels of CD4+ and CD8+ T lymphocytes, PaO_2_, PCO_2_, and ATP. The rats in the sepsis + saline group displayed ruptured alveolar walls and evident interstitial lung edema. However, the rats in the sepsis + remazolam group showed improved alveolar structure. Furthermore, the HMGB1 protein expression in the sepsis + remazolam group was lower than the sepsis + saline group.

**Conclusion:**

Remazolam can alleviate the inflammatory response in infected rats, thereby alleviating lung injury and improving immune function, which may be attributed to the reduction in HMGB1 protein expression.

## Introduction

Sepsis is a kind of fatal organ failure that induced by severe infection. One of the most common complications is acute respiratory distress syndrome (ARDS) (Dombrovskiy et al., 2007), which is the leading cause of death in the intensive care units (ICUs).

It has been reported that the serum levels of IL-1β and TNF-α in some sepsis patients are significantly increased, which is positively correlated with the inflammatory reaction and the severity of the disease (Zhen, Li & Wang, 2013). Although invasive mechanical ventilation, continuous renal replacement therapy (CRRT), extracorporeal membrane oxygenation (ECMO), and broad-spectrum powerful antibiotics are all used to sustain organ function in patients with sepsis and ARDS, their clinical efficacy is not promising. Currently, it is believed that ARDS is induced by excessive and unchecked inflammatory reactions in the lung tissue (Zhang et al., 2013).

Based on the pathophysiological basis of massive neutrophil exudation, ARDS is clinically characterized by pulmonary edema and refractory hypoxemia, resulting in extensive lung tissue damage (Mondrinos et al., 2013). Therefore, one of the essential treatments for the prevention and treatment of ARDS is to suppress proinflammatory and anti-inflammatory responses  (Kiemer, Muller & Vollmar, 2002). Remazolam, a brand-new ultrashort-acting benzodiazepine and water-soluble, has been extensively utilized to numb the pain and induce drowsiness in ICU. Remazolam has been reported to reduce inflammatory stress, but the potential mechanism remains unknown. In order to understand the protective effects of remazolam on lung tissue in septic rats with ARDS, we measured the levels of inflammatory markers and oxidative stress indicators in the lung tissue of septic rats.

## Materials and Methods

### Experimental animals

Forty male Sprague–Dawley (SD) rats (SPF grade, 8 weeks old) were provided by Haikou Yushi Biotechnology Co., Ltd. (Haikou, China). The number of experimental animals was determined according to previous studies and our pre-experimental results (Hong et al., 2022; Zhou, Wang & Zhang, 2018). Rats were maintained at a temperature of 20–26 °C and a relative humidity of 80%. The experiment was carried out after 1 week of adaptive feeding. Before operation, the animals fasted for 12 h without drinking water.

### Drugs and reagents

The reagents included Remazolam (No. 220608AU; Jiangsu Hengrui Pharmaceutical Co., Ltd., Nanjing, China); enzyme-linked immunosorbent assay (ELISA) kits (Shanghai Enzyme-linked Biotechnology Co., Ltd., Shanghai, China) of rat interleukin-6 (IL-6, No. ml064292), interleukin-1β (IL-1β, No. mIC50300-1), tumor necrosis factor-α (TNF-α, No. ml002859), IL-10 (NO. ml002813), procalcitonin (PCT, No. ml026011), and C reactive protein (CRP, No. ml027874), CD4 (No. ml038374), and CD8 (No. ml038372); malondialdehyde (MDA) test kit (No. E-EL-0060; Elabscience, Wuhan, China), myeloperoxidase (MPO) test kit (No. E-UNEL-H0048; Elabscience, Wuhan, China), adenosine triphosphate (ATP) test kit (No. E-BC-K831-M; Elabscience, Wuhan, China); and HMGB1 antibody (No. AF7020; Affinity, Melbourne, Australia). Blood Gas Analyzer was obtained from OPTI Medical Systems (Atlanta, GA, USA).

### Model preparation

Septic model was established by cecal ligation and puncture (CLP). Rats were anesthetized by intraperitoneal injection of 1% pentobarbital (40 mg/kg) and fixed after satisfactory anesthesia induction. After routine disinfection of abdominal skin and placement of sterile tissue, a 2-cm midline incision was made to open the abdominal cavity. The cecum was exposed, and ligated at its root. Puncture the cecum with an 18-gauge needle twice to form an intestinal fistula, and place two drainage strips to prevent the pinhole from closing. If no obvious bleeding occurred, put the cecum back into the abdominal cavity and suture the abdominal incision. In the sham operation + saline group and sham operation + remazolam group, the abdominal cavity was opened to expose the cecum, and pulled the cecum out, and put back into the abdominal cavity. Then suture the abdominal incision. After the operation, each rat was injected with 1 mL of normal saline intraperitoneally to avoid shock immediately. The rats then were free to eat and drink water after the operation. When an rat showed the abovementioned symptoms, it was given 1% pentobarbital (40 mg/kg) sodium by rapid intravenous injection to suppress the respiratory system so that rats stopped breathing and died. During our experiment, no unexpected adverse events occurred.

### Animal grouping

**Figure 1:**
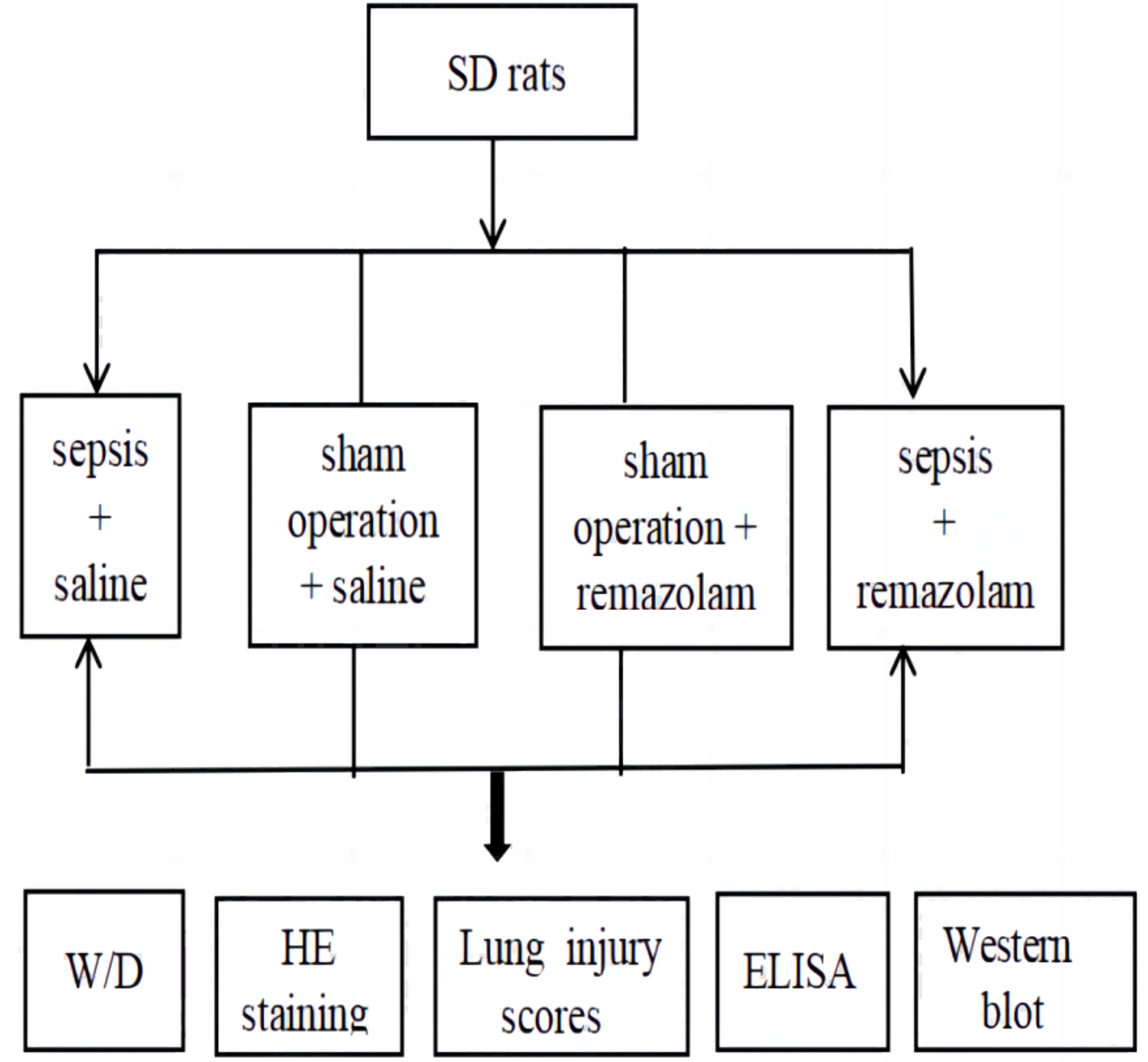
Experimental chart.

Forty rats were divided into four groups according to the random number table as follows (Fig. 1): the sepsis + saline group (*n* = 10), sham operation + saline group (*n* = 10), sham operation + remazolam group (*n* = 10), and sepsis + remazolam group (*n* = 10). All animal experimental protocols were approved by the Medical Ethics Committee of Hainan General Hospital (No. Med-Eth-Re[2022]630).

### Drug intervention

Rats in the sepsis + saline and sham operation + saline groups were intraperitoneally injected with saline (4 mL/kg) at 6, 12, 24, and 48 h after the procedure. Rats in the sham operation + remazolam and sepsis + remazolam groups were intraperitoneally injected with 6 mg/kg remazolam at 6, 12, 24, and 48 h after the procedure (according to literature, both intraperitoneal and intravenous injection can be used for drug intervention, but intraperitoneal injection is relatively fast and simple), and the dose was referred previous studies (Zhen, Huang & Li, 2021; Chen, Yang & Shi, 2023). Blood samples (2 mL) were collected from eyeballs under anesthesia at 6 h after operation, and heparin (10 U/mL) was used to anticoagulated. Serum samples were collected by centrifugation at 1006.2× g for 15 min at 4 °C. Rats were euthanized after blood collection, and the lung tissue was removed. Food and water consumption in each group was observed during saline/drug administration.

### Measurements

a. The right lung tissues of the rats were removed after euthanasia, surface moisture was removed, and the wet weight was measured. The lung tissue was placed into a 60 ° C-heating dry box for 48 h to constant weight, and the dry weight was measured. The ratio of wet weight to dry weight (W/D) was calculated.
b. Left lung tissues of the rats were fixed in 4% paraformaldehyde, embedded in conventional paraffin, sectioned, dewaxed and subjected to conventional hematoxylin-eosin (HE) staining, optical microscope observation and photographing.
c. For lung tissue injury scoring, with reference to the methods followed by Kiss et al. (2016) and Ozdulger et al. (2003), the presence of lung tissue edema, lung tissue structure damage, and neutrophil infiltration was scored, with one score indicating no injury, two indicating mild injury, three indicating moderate injury, and four indicating severe injury.
d. The levels of serum IL-6, TNF-α, IL-1β, IL-10, CD4+ and CD8+ T lymphocytes, MDP, MPO and ATP were measured by ELISA kits. The experimental operation steps referred to the ELISA kits.
e. Western blotting was used to detect the HMGB1 protein expression. After washing with PBS for 2–3 times, the lung tissue was cut into small pieces, homogenized, and extracted to collect the total protein. After making gel, sample separation and membrane transfer were performed. Finally, the WB results were observed and analyzed.

### Statistical analysis

Data are expressed as means ±standard deviation (SD). Analysis of variance was used to compare the differences among multiple groups. The least significant difference (LSD) *t* test was used for multiple comparisons. *P* < 0.05 was considered statistically significant. Statistical analysis was performed by GraphPad Prism v8.

## Results

**Figure 2:**
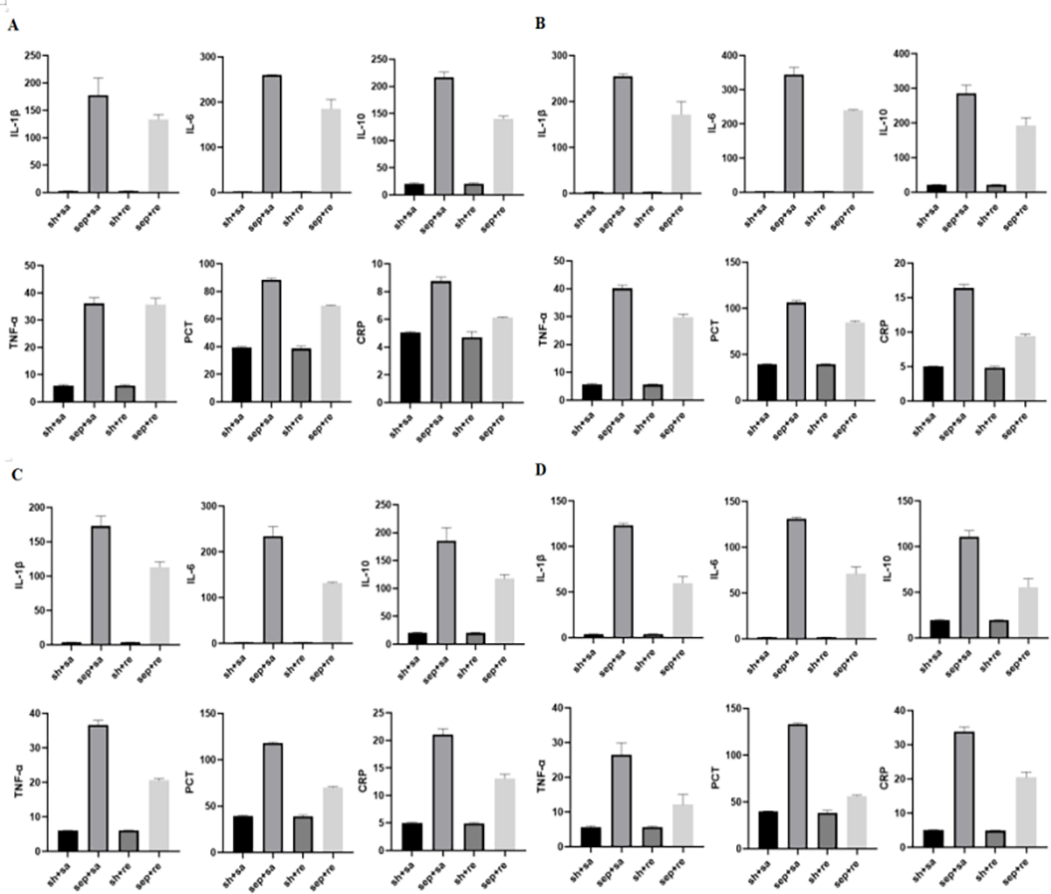
Expression of inflammatory factors in rats at different time periods. (A) Levels of inflammatory factors in rats after cecal perforation for 6 h; (B) levels of inflammatory factors in rats after cecal perforation for 12 h; (C) levels of inflammatory factors in rats after cecal perforation for 24 h; (D) levels of inflammatory factors in rats after cecal perforation for 48 h.

**Figure 3:**
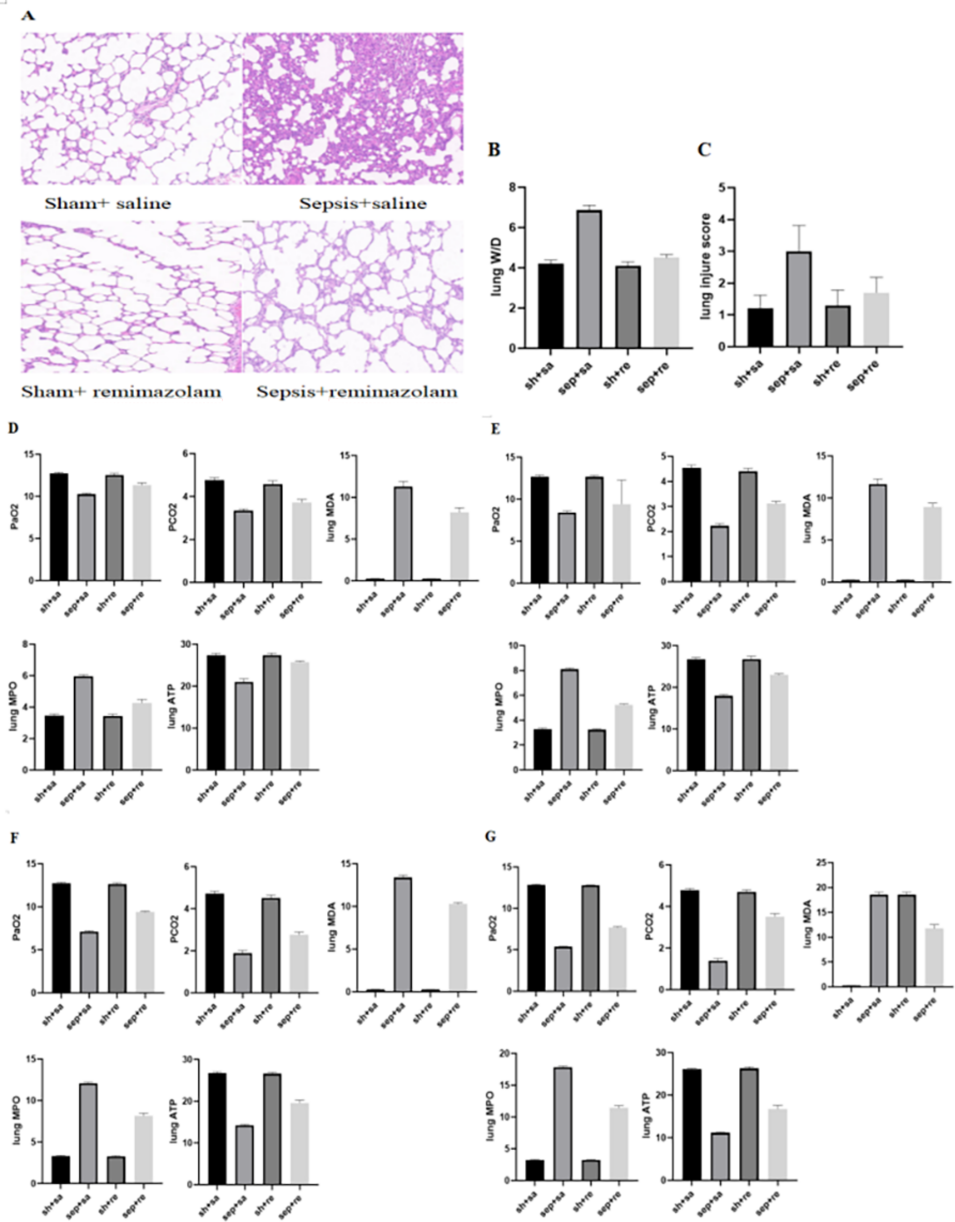
Changes in lung tissue parameters in each group. (A) Pathological manifestations of lung tissue; (B) lung W/D in each group; (C) lung injure score in each group; (D) blood gas analysis and changes of oxidative stress parameters for 6 h; (E) blood gas analysis and changes of oxidative stress parameters for 12 h; (F) blood gas analysis and changes of oxidative stress parameters for 24 h; (G) blood gas analysis and changes of oxidative stress parameters for 48 h.

After CLP, serum inflammatory factors were detected at 6, 12, 24 and 48 h. The levels of inflammatory factors began to increase after 6 h and gradually increased by time. The levels of inflammatory factors were obviously decreased in the sepsis + remazolam group in comparison with the sepsis + saline group. The W/D and lung injury scores of rats were increased in the sepsis + saline group, but decreased in the sepsis + remazolam group. There was no significant change in the levels of inflammatory factors between the sham operation + saline and sham operation + remazolam groups (Figs. 2, 3B and 3C, Tables 1 and 2).

**Table 1:** Expression of inflammatory factors in different time periods of rats in each group.

Group	6 h	P1	12 h	P2	24 h	P3	48 h	P4
IL-1β								
Sepsis + re	133.4 ± 8.88^**^	<0.0001	171.7 ± 27.84^**^	<0.0001	113.1 ± 7.58^**^	<0.0001	59.76 ± 7.29^**^	<0.0001
Sham + re	3.29 ± 0.10		3.53 ± 0.19		3.72 ± 0.17	<0.0001	3.93 ± 0.24	
Sham + saline	3.32 ± 0.17		3.59 ± 0.28		3.74 ± 0.13		3.97 ± 0.22	
Sepsis + saline	177.6 ± 31.64^*^	<0.0001	255.2 ± 5.05^*^	<0.0001	173.1 ± 14.33^*^		123.4 ± 2.00^*^	<0.0001
IL-6								
Sepsis + re	185.2 ± 20.71^**^	<0.0001	239.2 ± 2.28^**^	<0.0001	131.4 ± 2.61^**^	<0.0001	71.04 ± 7.61^**^	<0.0001
Sham + re	2.54 ± 0.17		3.06 ± 0.33		2.51 ± 0.22		1.90 ± 0.17	
Sham + saline	2.49 ± 0.22	<0.0001	3.156 ± 0.09	<0.0001	2.50 ± 0.18	<0.0001	1.88 ± 0.14	<0.0001
Sepsis + saline	260.2 ± 0.81^*^		343.9 ± 20.84^*^		234.0 ± 21.30^*^		131.1 ± 1.57^*^	
IL-10								
Sepsis + re	140.0 ± 5.18^**^	<0.0001	193.0 ± 21.81^**^	<0.0001	117.3 ± 7.48^**^	<0.0001	55.74 ± 8.93^**^	<0.0001
Sham + re	20.63 ± 1.73		21.55 ± 1.45		20.21 ± 0.99		19.71 ± 0.64	
Sham + saline	20.48 ± 1.99	<0.0001	21.27 ± 1.20	<0.0001	20.70 ± 1.23	<0.0001	19.76 ± 0.82	<0.0001
Sepsis + saline	216.7 ± 9.97^*^		285.4 ± 24.29^*^		185.6 ± 23.26^*^		110.9 ± 6.41^*^	
TNF-α								
Sepsis + re	35.65 ± 2.40^**^	<0.0001	29.76 ± 1.09^**^	<0.0001	20.57 ± 0.61^**^	<0.0001	12.13 ± 2.96^**^	<0.0001
Sham + re	6.04 ± 0.37		5.59 ± 0.22		6.04 ± 0.14		5.62 ± 0.29	
Sham + saline	6.06 ± 0.34	<0.0001	5.71 ± 0.30	<0.0001	6.05 ± 0.08	<0.0001	5.68 ± 0.36	<0.0001
Sepsis + saline	36.16 ± 2.06^*^		40.16 ± 1.11^*^		36.65 ± 1.37^*^		26.50 ± 3.43^*^	
PCT								
Sepsis + re	69.50 ± 0.53^**^	<0.0001	84.93 ± 1.26^**^	<0.0001	69.98 ± 1.23^**^	<0.0001	56.17 ± 1.33^**^	<0.0001
Sham + re	38.61 ± 1.86		39.25 ± 0.68		38.89 ± 1.76		38.38 ± 2.67	
Sham + saline	39.39 ± 0.79	<0.0001	39.51 ± 0.48	<0.0001	39.58 ± 0.79	<0.0001	39.67 ± 0.58	<0.0001
Sepsis + saline	88.38 ± 1.16^*^		106.4 ± 1.94^*^		118.0 ± 1.07^*^		133.3 ± 1.17^*^	
CRP								
Sepsis + re	6.12 ± 0.06^**^	<0.0001	9.42 ± 0.29^**^	<0.0001	13.10 ± 0.73^**^	<0.0001	20.47 ± 1.55^**^	<0.0001
Sham + re	4.70 ± 0.41		4.85 ± 0.20		4.91 ± 0.19		4.91 ± 0.10	
Sham + saline	5.07 ± 0.07	<0.0001	5.00 ± 0.09	<0.0001	5.03 ± 0.12	<0.0001	5.05 ± 0.09	<0.0001
Sepsis + saline	8.76 ± 0.27^*^		16.40 ± 0.48^*^		21.06 ± 1.07^*^		33.81 ± 1.41^*^	

**Notes.**

Expression of inflammatory factors in different time periods of rats in each group.

Compared with the sham + saline group, * <0.0001.

Compared with the sepsis + saline, ** <0.0001.

**Table 2:** Comparison of lung WD and lung injury score in each group of rats.

Group	Lung WD	P	Lung Injury Score	P
Sepsis + re	4.50 ± 0.16^**^	<0.0001	1.70 ± 0.48^**^	<0.0001
Sham + re	4.11 ± 0.19		1.30 ± 0.48	
Sham + saline	4.21 ± 0.18	<0.0001	1.20 ± 0.42	<0.0001
Sepsis + saline	6.87 ± 0.24^*^		3.00 ± 0.82^*^	

**Notes.**

Comparison of Lung WD and Lung Injury Score in each group of rats.

Compared with the sham + saline group, * <0.0001.

Compared with the sepsis + saline group, ** <0.0001.

Under the light microscope, the alveolar structure of rats were normal, and there was no inflammatory cell infiltration in the sham operation + saline and sham operation + remazolam groups. In the sepsis + saline group, the alveolar structure was destroyed, the alveolar wall was broken, pulmonary interstitial edema was obvious, and there were a large number of inflammatory cells, such as neutrophils, infiltrated. In the sepsis + remazolam group, the alveolar wall was relatively intact, the degree of pulmonary interstitial edema was reduced, and there were a small number of inflammatory cells infiltrated (Fig. 3A).

Compared with the sepsis + saline group, the oxygen partial pressure of blood gas analysis was increased, and carbon dioxide partial pressure of blood gas analysis was reduced in the sepsis + remazolam group (Figs. 3D–3G and Table 3).

**Table 3:** Blood gas analysis and expression levels of oxidative stress factors in the lung tissue of rats in each group.

Group	6 h	P1	12 h	P2	24 h	P3	48 h	P4
PaO2								
Sepsis + re	11.36 ± 0.24^**^	<0.0001	9.44 ± 2.84^**^	<0.0001	9.39 ± 0.11^**^	<0.0001	7.69 ± 0.12^**^	<0.0001
Sham + re	12.56 ± 0.19		12.67 ± 0.17		12.63 ± 0.17		12.8 ± 0.03	
Sham + saline	12.75 ± 0.13	<0.0001	12.69 ± 0.20	<0.0001	12.72 ± 0.16	<0.0001	12.86 ± 0.07	<0.0001
Sepsis + saline	10.26 ± 0.15^*^		8.42 ± 0.19^*^		7.11 ± 0.11^*^		5.38 ± 0.08^*^	
PCO2								
Sepsis + re	3.72 ± 0.14^**^	<0.0001	3.11 ± 0.09^**^	<0.0001	2.77 ± 0.13^**^	<0.0001	3.52 ± 0.14^**^	<0.0001
Sham + re	4.58 ± 0.15		4.41 ± 0.11		4.51 ± 0.14		4.71 ± 0.10	
Sham + saline	4.76 ± 0.11	<0.0001	4.54 ± 0.12	<0.0001	4.73 ± 0.12	<0.0001	4.78 ± 0.09	<0.0001
Sepsis + saline	3.35 ± 0.07^*^		2.23 ± 0.10^*^		1.89 ± 0.13^*^		1.39 ± 0.10^*^	
Lung MDA								
Sepsis + re	8.22 ± 0.54^**^	<0.0001	8.94 ± 0.44^**^	<0.0001	10.27 ± 0.18^**^	<0.0001	11.73 ± 0.78^**^	<0.0001
Sham + re	0.27 ± 0.009		0.27 ± 0.016		0.26 ± 0.009		0.26 ± 0.018	
Sham + saline	0.28 ± 0.02	<0.0001	0.28 ± 0.02	<0.0001	0.28 ± 0.02	<0.0001	0.28 ± 0.02	<0.0001
Sepsis + saline	11.30 ± 0.60^*^		11.66 ± 0.61^*^		13.40 ± 0.28^*^		18.55 ± 0.55^*^	
Lung MPO								
Sepsis + re	4.28 ± 0.20^**^	<0.0001	5.24 ± 0.07^**^	<0.0001	8.16 ± 0.30^**^	<0.0001	11.42 ± 0.38^**^	<0.0001
Sham + re	3.43 ± 0.15		3.24 ± 0.07		3.28 ± 0.10		3.24 ± 0.06	
Sham + saline	3.45 ± 0.13	<0.0001	3.27 ± 0.11	<0.0001	3.31 ± 0.10	<0.0001	3.23 ± 0.09	<0.0001
Sepsis + saline	5.97 ± 0.09^*^		8.09 ± 0.12^*^		12.06 ± 0.16^*^		17.84 ± 0.19^*^	
Lung ATP								
Sepsis + re	25.74 ± 0.24^**^	<0.0001	22.94 ± 0.33^**^	<0.0001	19.58 ± 0.67^**^	<0.0001	16.76 ± 0.87^**^	<0.0001
Sham + re	27.4 ± 0.55		26.73 ± 0.72		26.59 ± 0.36		22.26 ± 0.43	
Sham + saline	27.33 ± 0.53	<0.0001	26.71 ± 0.46	<0.0001	26.69 ± 0.43	<0.0001	26.11 ± 0.16	<0.0001
Sepsis + saline	20.98 ± 0.74^*^		17.95 ± 0.36^*^		14.27 ± 0.17^*^		11.18 ± 0.16^*^	

**Notes.**

Blood gas analysis and expression levels of oxidative stress factors in lung tissue of rats in each group.

Compared with the sham + saline, * <0.0001.

Compared with the sepsis + saline, ** <0.0001.

**Figure 4:**
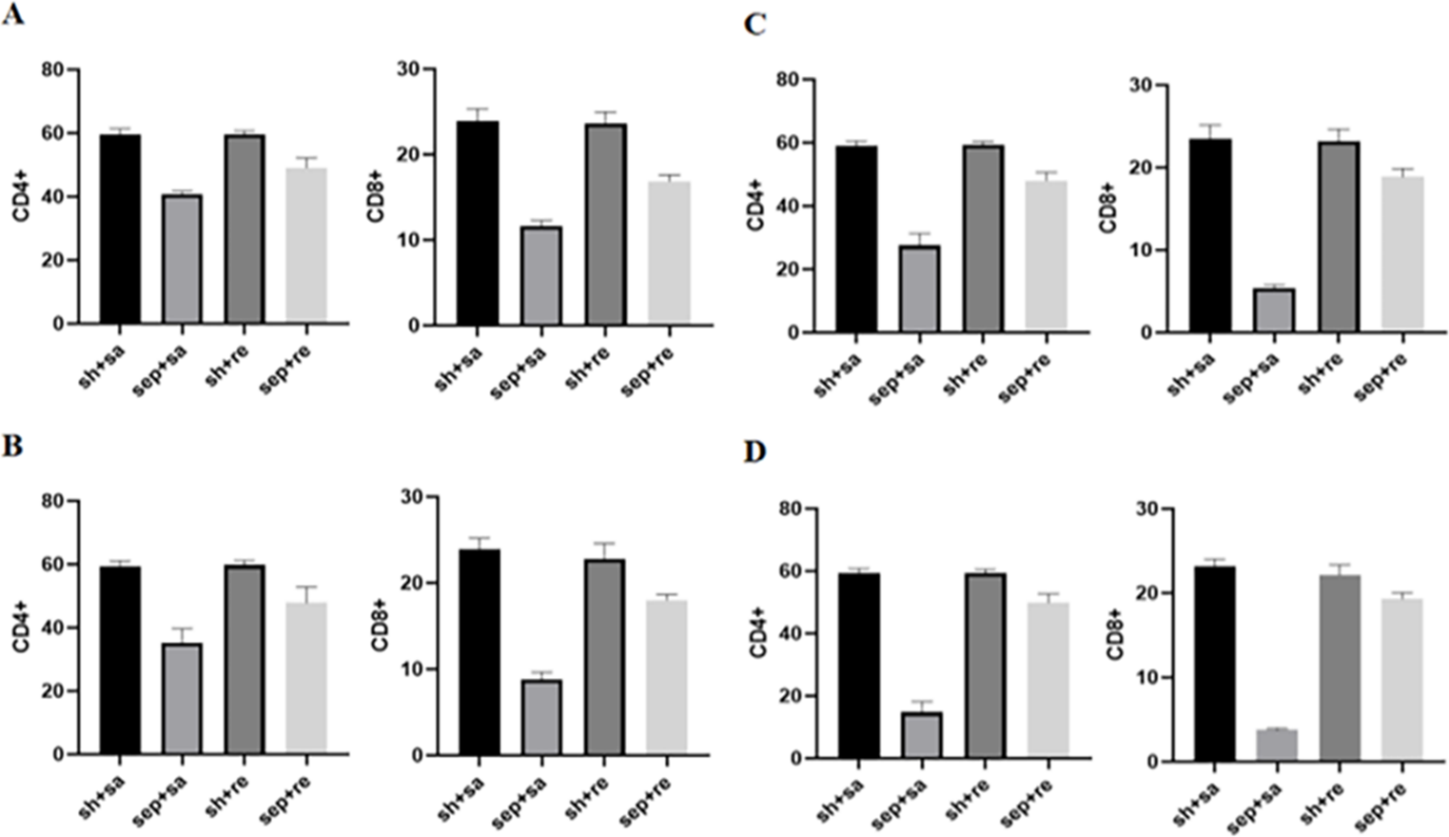
Changes in immune function indexes in each group. (A) Immune cell expression level for 6 h; (B) immune cell expression level for 12 h; (C) immune cell expression level for 24 h; (D) immune cell expression level for 48 h.

Compared with the sepsis + remazolam group, the contents of CD4+ and CD8+ T cells in the sepsis + saline were decreased, but there was no significant change between the sham operation + saline and sham operation + remazolam groups (Fig. 4 and Table 4).

Compared with the sham operation + saline and sham operation + remazolam groups, the levels of MDP and MPO were significantly higher in the sepsis + saline group, while ATP level was lower, and the opposite was true in the sepsis + remazolam group (Figs. 3D–3G, Table 3).

**Figure 5:**
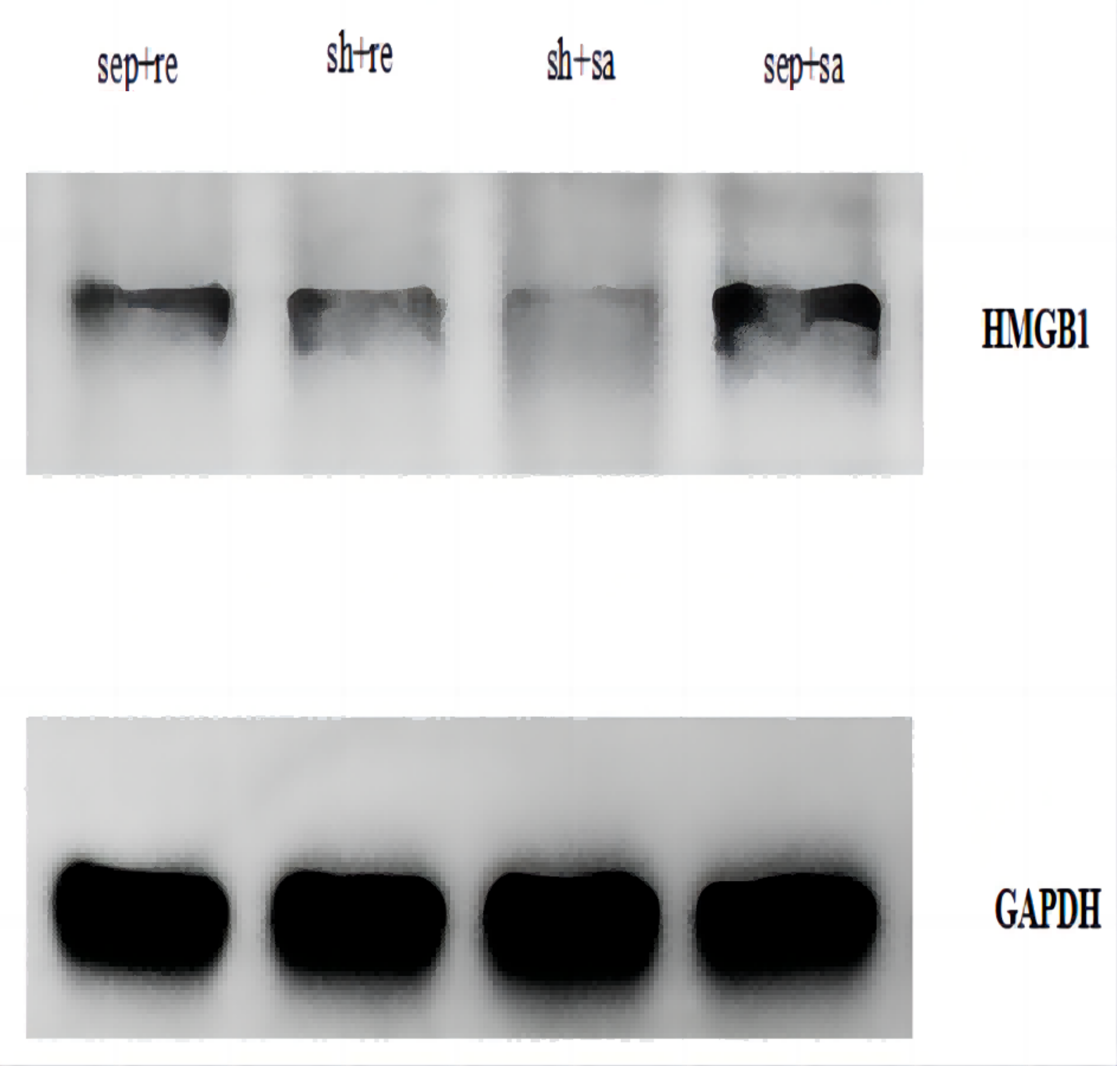
Expression of HMGB1 protein in lung tissue of each group.

The HMGB1 protein expression was significantly increased in the sepsis + saline group but decreased in the sepsis + remazolam group (Fig. 5).

## Discussion

ARDS is an acute inflammatory injury that causes by extrapulmonary or intrapulmonary factors, and the morbidity and mortality increase significantly belong the aggravation of sepsis (Bellani et al., 2016). The symptoms of ARDS patients will be more serious if associated with sepsis, and have poor prognosis and higher mortality (Sheu et al., 2010). At present, the pathogenesis of ARDS remains unclear, but more and more studies believe that systemic inflammatory response plays a key role at the beginning and development process of ARDS (Jiang et al., 2018; Yu et al., 2015). In conclusion, inhibiting the production of inflammatory factors may be an effective way to cure ARDS. From our experiment, we found that proinflammatory factors, such as IL-1 β, IL-6, and TNF-α, in peripheral serum of septic rats were obviously increased at 6 h, and anti-inflammatory factor, IL-10, was decreased, which further confirming that proinflammatory and anti-inflammatory systems of the body are in an unbalanced state during sepsis. Under the light microscope, a large number of inflammatory cells infiltrated into the alveolar wall and interstitial lung of rats in the sepsis + saline group, and the alveolar structure was broken and destroyed. Combined with W/D of lung tissue and lung injury score, these results proved that the ARDS model induced by cecal perforation was successful. By comparing the serum inflammatory factors in rats in each group, we found that the levels of inflammatory mediators in the circulating serum of rats decreased obviously, the structure of lung tissue, infiltration of inflammatory cells also improved obviously, partial pressure of arterial oxygen in arterial blood gas increased, and partial pressure of carbon dioxide decreased after drug intervention with remazolam. It indicated that remazolam might reduce lung tissue damage, improve oxygenation capacity and alleviate ARDS induced by sepsis while inhibiting systemic inflammatory reactions, but specific mechanism is unclear.

**Table 4:** Expression levels of immune factors in rats of each group.

Group	6 h	P1	12 h	P2	24 h	P3	48 h	P4
CD4+								
Sepsis + re	49.10 ± 3.11^**^	<0.0001	47.84 ± 4.98^**^	<0.0001	48.03 ± 2.57^**^	<0.0001	49.81 ± 2.88^**^	<0.0001
Sham + re	59.66 ± 1.11		59.80 ± 1.47		59.34 ± 1.04		59.41 ± 1.15	
Sham + saline	59.53 ± 1.83		59.39 ± 1.69		58.98 ± 1.59		59.33 ± 1.54	
Sepsis + saline	40.85 ± 0.87^*^	<0.0001	35.28 ± 4.45^*^	<0.0001	27.53 ± 3.73^*^	<0.0001	14.78 ± 3.39^*^	<0.0001
CD8+								
Sepsis + re	16.86 ± 0.75^**^	<0.0001	18.01 ± 0.64^**^	<0.0001	18.89 ± 0.99^**^	<0.0001	19.31 ± 0.74^**^	<0.0001
Sham + re	23.58 ± 1.37		22.77 ± 1.83		23.20 ± 1.49		22.15 ± 1.20	
Sham + saline	23.92 ± 1.42	<0.0001	23.89 ± 1.31	<0.0001	23.48 ± 1.69	<0.0001	23.18 ± 0.83	
Sepsis + saline	11.61 ± 0.67^*^		8.82 ± 0.85^*^		5.37 ± 0.43^*^		3.76 ± 0.22^*^	<0.0001

**Notes.**

Expression levels of immune factors in rats of each group.

Compared with the sham + saline, * <0.0001.

Compared with the sepsis + saline, ** <0.0001.

Immune regulation dysfunction also be one of the important mechanisms leading to the progression of sepsis. Important immune cells in sepsis patients are missing  (Tinsley et al., 2001; Hotchkiss & Tinsley, 2002). Types of missing immune cells include CD4 T and CD8 T cells. CD8+ T lymphocytes regulate activation and proliferation of CD4+ T lymphocytes in cell–cell contact-dependent manner (Elrefaei et al., 2007). In the advanced stage of sepsis, CD4+ T cells, CD8+ T cells and monocytes are reduced to varying degrees, and immunosuppression is aggravated (Riedemann, Guo & Ward, 2003; Barochia et al., 2010), which increases the severity of the disease (Xiufen, Shunyao & Qingming, 2018). By measuring CD4+ and CD8+ T lymphocytes in serum of rats, we found that the number of these lymphocytes in septic rats decreased obviously, and the downward trend was more obvious by increasing inflammatory factors, which may lead to the apoptosis of immune cells and inhibit immune function.

In the process of sepsis, oxidative stress injury is another aspect that needs to be considered. The imbalance of oxidation/antioxidation in the lung plays an important role in ARDS (Barabas et al., 2013; Jing, Cuili & Zhiguo, 2014; Qin et al., 2018). The contents of MDA and MPO can predict the level of lipid peroxide (Tariq et al., 2003). We found that contents of MDA and MPO in the sepsis + saline group were significantly higher than the sepsis + remazolam group, suggesting that remazolam reduces the degree of lipid peroxidation and production of oxygen free radicals. In addition, the ATP level in the sepsis + saline group was lower than the sepsis + remazolam group, indicating that all tissues and organs in the whole body are in a state of high metabolism during sepsis, but there is ischemia and hypoxia due to circulatory stagnation and hypoperfusion, which leads to disorder of cellular energy metabolism. The mechanism may be related to sodium-potassium pump that relies on ATP for energy supply. After the intervention of remazolam, ATP synthesis increased and lung tissue structure damage was improved, indicating that it has a regulatory effect on the balance of oxygen supply and high metabolism in lung tissue.

In addition, we also observed the HMGB1 protein expression in each group. HMGB1 is an important late inflammatory mediator (Yang, Wang & Tracey, 2001), and it plays a key regulatory role in the inflammatory response network of sepsis. Fang et al. (2002) found that compared with TNF-α, IL-1β and other relatively early cytokines, HMGB1 appeared later but lasted longer. The rat model of sepsis established by CLP found that increasing serum HMGB1 level was related to organ dysfunction (Ma et al., 2009). Our results showed that HMGB 1 was expressed in each group, but the expression in the sepsis + saline group was increased, because HMGB1 is a ubiquitous protein that can be slightly expressed in normal cells, and can be upregulated by infection. In our experiment, we also found that severe sepsis in rats is in direct proportion to the HMGB1 expression, which became weakened after treatment with remazolam. It shows that remazolam has a certain inhibitory effect on HMGB1, playing an anti-inflammatory and antioxidative stress role and improving cellular immune function. However, the deficiency of the study is that we did not observe the concentration gradient of remazolam, nor did we observe multiple time points during sepsis. We will design future research base on different doses of remazolam and more intervention time points for comparative experiments to explore the protective mechanism of remazolam on septic ARDS, and potential signal pathways and target genes are also needed to explore to provide more experimental basic data and theoretical basis for clinical application.

## Supplemental Information

10.7717/peerj.17205/supp-1 Supplemental Information 1 Author Checklist

10.7717/peerj.17205/supp-2 Supplemental Information 2 Blots

## Additional Information and Declarations

### Data Availability

The following information was supplied regarding data availability: The data is available at Figshare: Xiaoxin, Gao (2023). sepsis1. figshare. Dataset. https://doi.org/10.6084/m9.figshare.22638901.v1. Xiaoxin, Gao (2022). data1. figshare. Dataset. https://doi.org/10.6084/m9.figshare.21587649.v1. Xiaoxin, Gao (2022). data2. figshare. Figure. https://doi.org/10.6084/m9.figshare.21587661.v1. Xiaoxin, Gao (2023). experience datas. figshare. Dataset. https://doi.org/10.6084/m9.figshare.23522562.v1. The uncropped blots are available in the Supplemental File.
