# Supplementary figures and images for: Preliminary study on the protective effect of remazolam against sepsis-induced acute respiratory distress syndrome (ARDS)
Source: PeerJ. 2024 Apr 18;12:e17205. doi: 10.7717/peerj.17205 (PMC11032653; doi:10.7717/peerj.17205)

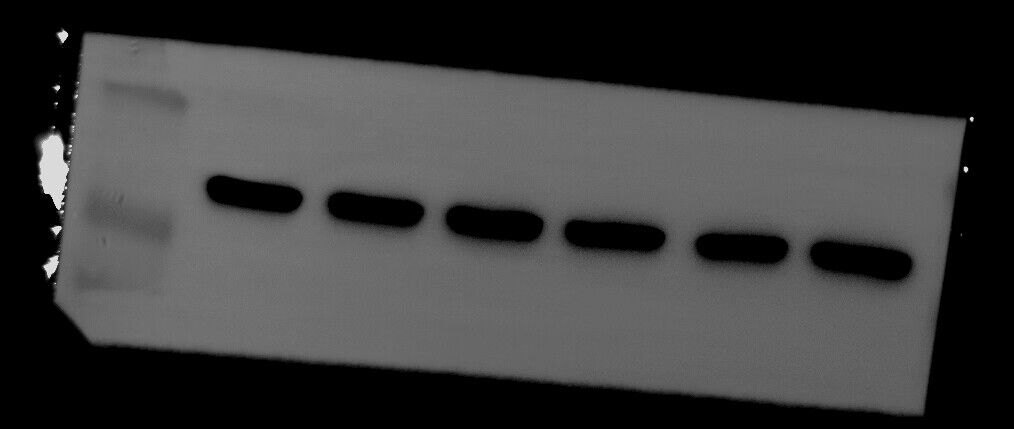

Supplement: Supplemental Information 2 [file peerj-12-17205-s002.zip › Figure 4 uncroped 2.jpg]

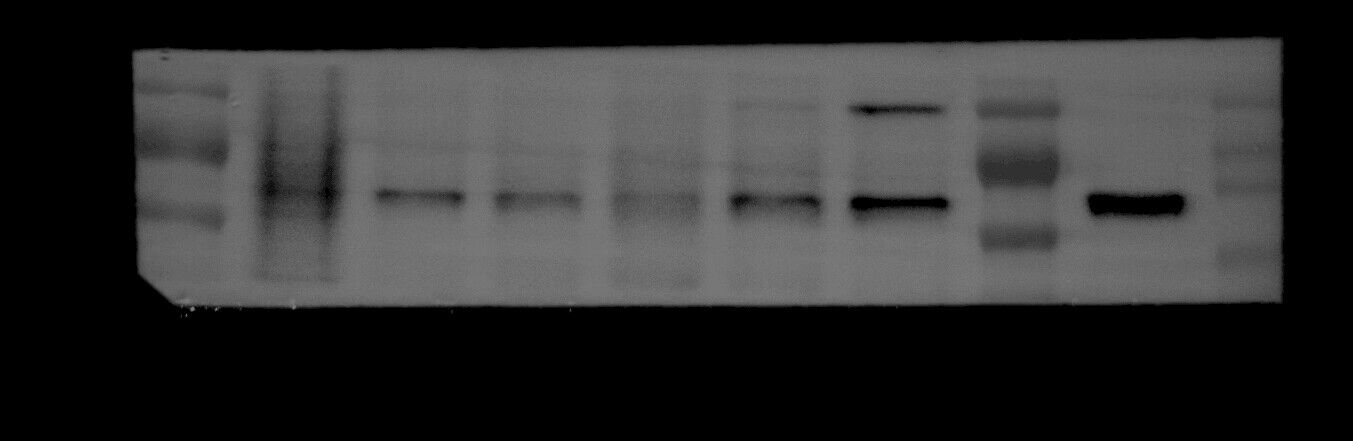

Supplement: Supplemental Information 2 [file peerj-12-17205-s002.zip › Figure 4 uncroped 1.jpg]
